# Supplementary material for: Different HCV Exposure Drives Specific miRNA Profile in PBMCs of HIV Patients
Source: Biomedicines. 2021 Nov 5;9(11):1627. doi: 10.3390/biomedicines9111627 (PMC8615810; doi:10.3390/biomedicines9111627)
Supplement: Supplementary file 1 [file biomedicines-09-01627-s001.zip › biomedicines-1404144-supplementary.pdf]

1. Supplementary Materials and Methods

1.1 Patients

Exclusion criteria: pregnancy, individuals below 18 years old, previous HCV treatment, advanced liver fibrosis (>F3), clinical evidence of hepatic decompensation, active drug or alcohol addiction, alcohol-induced liver injury, HBV active infection, opportunistic infections, and other concomitant diseases such as diabetes, nephropathies, autoimmune disease, hemochromatosis, cryoglobulinemia, primary biliary cirrhosis, Wilson´s disease, α1-antitrypsin deficiency, and neoplasia.

Clinical records: Clinical and epidemiological data were obtained from medical records: HCV-RNA viral load (IU/mL). In the case of patients with a previous history of intravenous drug use (IDU), the time of infection was estimated since the first year they shared needles and other injection paraphernalia [1]. The liver stiffness measurement (LSM) was assessed by transient elastometry (FibroScan®, Echosens, Paris, France) and expressed in kilopascals (kPa). Subjects were stratified according to cut-offs of LSM: <7.1 kPa (F0–F1: absence or mild fibrosis) and 7.1–9.4 kPa (F2: significant fibrosis). The clinical characteristics of metabolic status and biochemical parameter of liver function and genotype of rs12979860 polymorphism at interferon lambda 4 (gene/pseudogene) (*IFNL4*) was also recorded.

1.2 High throughput sequencing of small RNA

Peripheral venous blood samples were collected in EDTA tubes, and PBMCs were isolated within the first 4 hours after extraction. Total RNA including miRNAs were isolated from PBMCs with the miRNeasy Mini kit (Qiagen). Quality and integrity of the RNA were evaluated by the Bioanalyzer 2100 with Agilent RNA 6000 Nano kit (Agilent). Only those samples with a RNA integrity number (RIN) > 7.5 were sequenced.

Small RNA library synthesis and sequencing were performed at Centre for Genomic Regulation (CRG) at Barcelona (Spain), as previously described [2]. Small RNA libraries were constructed with Illumina's TruSeq Small RNA kit v.4 (Illumina) and sequenced in an Illumina HiSeq2500, with 1x50.

1.3 MicroRNA data processing: Bioinformatics analysis

The raw data were initially filtered out for reads with ambiguous base calls. Quality control of the remaining sequences was performed by using FastQC (v0.11.3) [3]. Adapter sequences, as well as low quality base calls (q < 20), were trimmed with cutadapt (v. 1.18) [4]. Adapter trimmed reads were processed with miRDeep2 (v. 0.0.7) [5]. Only the alignments with 0 mismatches in the seed region and that do not map to more than five different loci in the genome were retained. miRBase v21 which contains 2888 precursors was used as reference.

1.4 Bibliografía

1. Thorpe, L. E.; Ouellet, L. J.; Hershow, R.; Bailey, S. L.; Williams, I. T.; Williamson, J.; Monterroso, E. R.; Garfein, R. S., Risk of hepatitis C virus infection among young adult injection drug users who share injection equipment. *Am. J. Epidemiol.* **2002,** *155* (7), 645-53.

2. Brochado-Kith, Ó.; Gómez Sanz, A.; Real, L. M.; Crespo García, J.; Ryan Murúa, P.; Macías, J.; Cabezas González, J.; Troya, J.; Pineda, J. A.; Arias Loste, M. T.; Díez Viñas, V.; Jiménez-Sousa, M.; Medrano de Dios, L. M.; Cuesta De la Plaza, I.; Monzón Fernández, S.; Resino García, S.; Fernández-Rodríguez, A., MicroRNA Profile of HCV Spontaneous Clarified Individuals, Denotes Previous HCV Infection. *J Clin Med* **2019,** *8* (6).

3. S., A. FastQC: a quality control tool for high throughput sequence data. http://www.bioinformatics.babraham.ac.uk/projects/fastqc.

4. Martin, M., Cutadapt removes adapter sequences from high-throughput sequencing reads. *EMBnet. journal* **2011,** *17* (1), 10-12.

5. Friedländer, M. R.; Mackowiak, S. D.; Li, N.; Chen, W.; Rajewsky, N., miRDeep2 accurately identifies known and hundreds of novel microRNA genes in seven animal clades. *Nucleic Acids Res* **2012,** *40* (1), 37-52.

2. Supplementary Figures

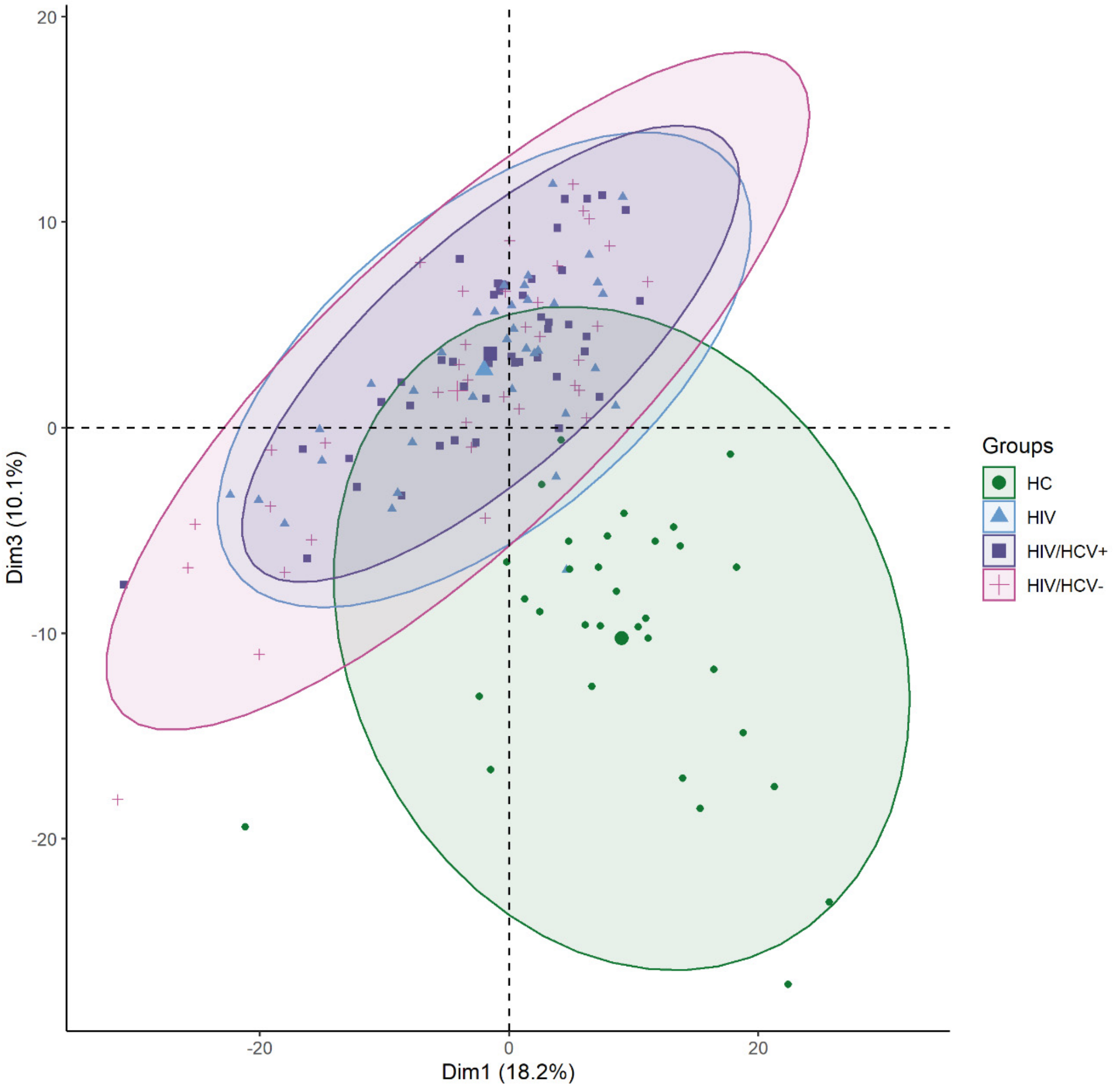

Figure S1. Partial least squares discriminant analysis (PLS-DA) regression of miRNA normalized expression data. PLS-DA method has been used to discriminate between patients in the HIV groups and healthy controls (HC). Each symbol represent the miRNA profile of PBMCs samples in HIV/HCV+ (purple), HIV/HCV- (pink), HIV (blue) and HC (green) patients. Symbols in bold represent the average of its group.

3. Supplementary Tables

Table S1. Differentially expressed miRNAs in HIV/HCV+ patients.

Table S2. MicroRNA-gene target enrichment analysis.

Table S3. Pathway enrichment analysis.

Table S4. Differentially expressed miRNAs in HIV/HCV- patients.

Table S5. Differentially expressed miRNAs in HIV patients.

Table S6. Top enriched target genes for specific miRNA deregulation signature in HIV/HCV+ patients.

Table S7. Top enriched target genes for specific miRNAs deregulation signature in HIV/HCV- patients exclusively.

Table S8. Top enriched target genes for specific miRNAs deregulation signature in HIV patients exclusively

Table S1. Differentially expressed miRNAs in HIV/HCV+ patients. Significant differentially expressed (SDE) miRNAs in HIV/HCV+ patients compared to healthy controls**.** Each column represent: the SDE miRNA identifier (miRBase format), the Log2FC indicating the expression fold change between HIV/HCV+ and healthy patients, and the adjusted p-value (FDR, corrected for multiple testing with the Benjamin–Hochberg procedure).

miRNAs	LogFC	FDR
hsa-miR-101-5p	-0,719	0
hsa-miR-10395-3p	-1,18	0
hsa-miR-10399-3p	0,703	0
hsa-miR-103a-2-5p	0,608	0,014
hsa-miR-10401-3p	-2,311	0
hsa-miR-11400	0,901	0
hsa-miR-12135	-0,85	0
hsa-miR-122-5p	-1,581	0
hsa-miR-1246	-1,323	0,004
hsa-miR-1248	-3,185	0
hsa-miR-1249-3p	-1,513	0
hsa-miR-125a-5p	-1,289	0
hsa-miR-125b-2-3p	-0,692	0,002
hsa-miR-125b-5p	-1,473	0
hsa-miR-126-5p	0,667	0,001
hsa-miR-1260a	-1,132	0
hsa-miR-1260b	-1,118	0
hsa-miR-1268a	-0,671	0
hsa-miR-127-3p	0,775	0
hsa-miR-127-5p	0,635	0,018
hsa-miR-1275	-0,635	0

hsa-miR-1277-5p	-0,626	0,004
hsa-miR-1285-3p	-0,849	0
hsa-miR-1291	-0,783	0
hsa-miR-1306-5p	-1,254	0
hsa-miR-1307-5p	-0,828	0,001
hsa-miR-132-5p	-0,762	0
hsa-miR-134-5p	0,86	0
hsa-miR-1343-3p	-0,9	0
hsa-miR-136-3p	0,608	0,025
hsa-miR-138-5p	-0,639	0,001
hsa-miR-139-5p	1,189	0
hsa-miR-142-3p	-0,791	0
hsa-miR-143-3p	0,901	0
hsa-miR-146a-5p	1,538	0
hsa-miR-146b-5p	1,217	0
hsa-miR-148a-5p	0,962	0
hsa-miR-148b-3p	0,752	0
hsa-miR-148b-5p	0,624	0
hsa-miR-150-5p	-1,297	0
hsa-miR-151a-3p	0,592	0
hsa-miR-152-5p	0,649	0,004
hsa-miR-154-5p	1,11	0
hsa-miR-181b-2-3p	-0,602	0,01
hsa-miR-1908-5p	-0,725	0
hsa-miR-193a-3p	-0,961	0
hsa-miR-193b-3p	-1,099	0
hsa-miR-196a-5p	0,864	0,002
hsa-miR-197-3p	-0,751	0
hsa-miR-204-5p	0,745	0,015
hsa-miR-20a-3p	-0,751	0
hsa-miR-21-3p	1,144	0
hsa-miR-210-5p	-1,625	0
hsa-miR-2110	-0,689	0
hsa-miR-23a-5p	-1,226	0
hsa-miR-24-3p	0,617	0
hsa-miR-2467-5p	1,057	0
hsa-miR-25-5p	-1,052	0
hsa-miR-296-5p	-1,701	0
hsa-miR-299-3p	0,666	0,033
hsa-miR-29a-5p	-0,797	0
hsa-miR-29c-3p	-0,705	0
hsa-miR-30a-5p	0,647	0
hsa-miR-31-5p	-0,592	0,004
hsa-miR-3142	-1,827	0
hsa-miR-3176	0,625	0,001
hsa-miR-3196	-1,477	0

hsa-miR-32-3p	-0,656	0
hsa-miR-32-5p	-0,778	0
hsa-miR-320c	-0,639	0
hsa-miR-320d	-0,616	0
hsa-miR-324-5p	0,604	0
hsa-miR-328-3p	-0,725	0
hsa-miR-329-3p	0,615	0,041
hsa-miR-331-3p	-0,79	0
hsa-miR-339-3p	-0,631	0
hsa-miR-33a-5p	-0,737	0
hsa-miR-342-5p	-0,942	0
hsa-miR-34a-5p	0,602	0,001
hsa-miR-3605-3p	-0,98	0
hsa-miR-3609	-1,979	0
hsa-miR-361-3p	-0,636	0
hsa-miR-3614-5p	0,988	0
hsa-miR-362-3p	-0,845	0
hsa-miR-362-5p	1,3	0
hsa-miR-3651	-0,99	0
hsa-miR-3677-3p	1,334	0
hsa-miR-3690	-0,653	0,002
hsa-miR-376a-3p	0,695	0,019
hsa-miR-379-5p	1,252	0
hsa-miR-381-3p	0,843	0,001
hsa-miR-382-3p	1,233	0
hsa-miR-3960	-2,591	0
hsa-miR-410-3p	0,86	0
hsa-miR-411-5p	0,941	0
hsa-miR-412-5p	1,881	0
hsa-miR-423-3p	-0,631	0
hsa-miR-423-5p	-1,081	0
hsa-miR-432-5p	0,635	0,012
hsa-miR-4433b-3p	-1,009	0,002
hsa-miR-4433b-5p	-1,364	0
hsa-miR-4454	-1,23	0
hsa-miR-4508	-2,586	0
hsa-miR-451a	0,934	0,024
hsa-miR-4531	0,623	0,006
hsa-miR-4645-3p	1,016	0
hsa-miR-4687-5p	-1,494	0
hsa-miR-4772-3p	-1,188	0
hsa-miR-4772-5p	-1,084	0
hsa-miR-485-5p	-0,62	0,019
hsa-miR-486-3p	-1,047	0
hsa-miR-493-3p	0,74	0,002
hsa-miR-493-5p	0,739	0,002

hsa-miR-495-3p	0,655	0,016
hsa-miR-496	1,057	0
hsa-miR-502-3p	-0,704	0
hsa-miR-505-5p	-1,38	0
hsa-miR-5100	-0,933	0
hsa-miR-5193	-0,616	0,002
hsa-miR-532-3p	-0,629	0
hsa-miR-548ad-5p	-0,644	0
hsa-miR-548ae-5p	-0,749	0
hsa-miR-548au-5p	-0,734	0
hsa-miR-548ax	-0,863	0
hsa-miR-548ay-5p	-0,714	0
hsa-miR-5701	-1,173	0
hsa-miR-582-3p	0,86	0
hsa-miR-582-5p	0,594	0,001
hsa-miR-589-3p	-0,729	0
hsa-miR-589-5p	0,675	0
hsa-miR-590-3p	-0,998	0
hsa-miR-624-5p	-0,814	0
hsa-miR-629-5p	0,848	0
hsa-miR-642a-3p	-0,898	0
hsa-miR-642a-5p	-0,688	0,001
hsa-miR-6511a-3p	-0,842	0
hsa-miR-6511b-3p	-1,24	0
hsa-miR-654-5p	0,793	0,001
hsa-miR-664b-5p	-0,638	0,001
hsa-miR-6724-5p	-0,836	0
hsa-miR-6803-3p	-1,577	0
hsa-miR-6837-3p	0,909	0
hsa-miR-7-5p	1,035	0
hsa-miR-7704	-1,334	0
hsa-miR-7976	-0,726	0
hsa-miR-7977	-1,299	0
hsa-miR-877-5p	-1,194	0
hsa-miR-889-3p	0,72	0,006
hsa-miR-9-5p	0,719	0
hsa-miR-95-3p	-0,65	0
hsa-miR-98-5p	0,653	0
hsa-miR-9985	-0,926	0
hsa-miR-99a-5p	-1,009	0

Table S2. MicroRNA-gene target enrichment analysis. Target-enrichment analysis for the SDE miRNAs found in each comparison (HIV/HCV+ vs HC, HIV/HCV- vs HC, and HIV vs HC). Here we have included the target gene symbol (Gene_symbol), the adjusted p-value (FDR, corrected p-value for multiple testing performed with the Benjamin–Hochberg procedure), the number of SDE miRNAs that interact each enriched gene (Num_interactions), and the miRNA identifier in miRBase format.

Analysis	Gene_symbol	FDR	Num_interactions	miRNAs
HIV/HCV +	RPS2	0,002	10	hsa-miR-423-5p / hsa-miR-361-3p / hsa-miR-25-5p / hsa-miR-423-3p / hsa-miR-324-5p / hsa-miR-331-3p / hsa-miR-328-3p / hsa-miR-125a-5p / hsa-miR-34a-5p / hsa-miR-196a-5p
HIV/HCV +	BCL11A	0,003	8	hsa-miR-148b-3p / hsa-miR-29c-3p / hsa-miR-196a-5p / hsa-miR-32-5p / hsa-miR-486-3p / hsa-miR-30a-5p / hsa-miR-2110 / hsa-miR-138-5p
HIV/HCV +	MEGF9	0,003	6	hsa-miR-151a-3p / hsa-miR-877-5p / hsa-miR-197-3p / hsa-miR-125b-5p / hsa-miR-125a-5p / hsa-miR-432-5p
HIV/HCV +	COPA	0,004	10	hsa-miR-193b-3p / hsa-miR-423-3p / hsa-miR-1260b / hsa-miR-127-5p / hsa-miR-376a-3p / hsa-miR-142-3p / hsa-miR-146b-5p / hsa-miR-146a-5p / hsa-miR-589-5p / hsa-miR-122-5p
HIV/HCV +	NFKB2	0,006	4	hsa-miR-193b-3p / hsa-miR-98-5p / hsa-miR-423-5p / hsa-miR-143-3p
HIV/HCV +	TGM2	0,006	5	hsa-miR-1285-3p / hsa-miR-34a-5p / hsa-miR-7-5p / hsa-miR-30a-5p / hsa-miR-127-3p
HIV/HCV +	THAP12	0,006	4	hsa-miR-34a-5p / hsa-miR-7-5p / hsa-miR-98-5p / hsa-miR-24-3p
HIV/HCV +	NFKB1	0,01	7	hsa-miR-9-5p / hsa-miR-146a-5p / hsa-miR-146b-5p / hsa-miR-139-5p / hsa-miR-138-5p / hsa-miR-339-3p / hsa-miR-34a-5p
HIV/HCV +	ITGBL1	0,013	7	hsa-miR-146a-5p / hsa-miR-362-3p / hsa-miR-329-3p / hsa-miR-889-3p / hsa-miR-20a-3p / hsa-miR-382-3p / hsa-miR-33a-5p
HIV/HCV +	MDN1	0,013	7	hsa-miR-642a-5p / hsa-miR-1260b / hsa-miR-146b-5p / hsa-miR-146a-5p / hsa-miR-589-5p / hsa-miR-362-3p / hsa-miR-329-3p
HIV/HCV +	PIK3CG	0,013	10	hsa-miR-148b-3p / hsa-miR-7-5p / hsa-miR-142-3p / hsa-miR-1277-5p / hsa-miR-362-3p / hsa-miR-329-3p / hsa-miR-410-3p / hsa-miR-127-3p / hsa-miR-34a-5p / hsa-miR-125a-5p
HIV/HCV +	NDE1	0,013	7	hsa-miR-30a-5p / hsa-miR-877-5p / hsa-miR-423-5p / hsa-miR-423-3p / hsa-miR-151a-3p / hsa-miR-99a-5p / hsa-miR-3196
HIV/HCV +	DDX56	0,013	4	hsa-miR-34a-5p / hsa-miR-423-3p / hsa-miR-331-3p / hsa-miR-328-3p
HIV/HCV +	PRKCH	0,013	7	hsa-miR-432-5p / hsa-miR-376a-3p / hsa-miR-493-3p / hsa-miR-4531 / hsa-miR-1343-3p / hsa-miR-3690 / hsa-miR-24-3p
HIV/HCV +	CASC4	0,013	4	hsa-miR-151a-3p / hsa-miR-125a-5p / hsa-miR-381-3p / hsa-miR-485-5p
HIV/HCV +	MCL1	0,023	16	hsa-miR-193a-3p / hsa-miR-29c-3p / hsa-miR-193b-3p / hsa-miR-582-5p /

				hsa-miR-148b-5p / hsa-miR-125b-5p / hsa-miR-151a-3p / hsa-miR-139-5p / hsa-miR-495-3p / hsa-miR-5193 / hsa-miR-32-5p / hsa-miR-125a-5p / hsa-miR-34a-5p / hsa-miR-339-3p / hsa-miR-320c / hsa-miR-320d
HIV/HCV +	ATP5B	0,023	7	hsa-miR-193b-3p / hsa-miR-423-5p / hsa-miR-339-3p / hsa-miR-324-5p / hsa-miR-331-3p / hsa-miR-125b-5p / hsa-miR-24-3p
HIV/HCV +	TLR2	0,024	5	hsa-miR-146a-5p / hsa-miR-154-5p / hsa-miR-101-5p / hsa-miR-143-3p / hsa-miR-23a-5p
HIV/HCV +	TRPM3	0,024	5	hsa-miR-204-5p / hsa-miR-4433b-5p / hsa-miR-1260b / hsa-miR-1260a / hsa-miR-532-3p
HIV/HCV +	POLD1	0,027	4	hsa-miR-24-3p / hsa-miR-193b-3p / hsa-miR-34a-5p / hsa-miR-324-5p
HIV/HCV +	HOTAIR	0,027	4	hsa-miR-34a-5p / hsa-miR-331-3p / hsa-miR-148b-3p / hsa-miR-126-5p
HIV/HCV +	SPTBN1	0,028	6	hsa-miR-9-5p / hsa-miR-34a-5p / hsa-miR-589-3p / hsa-miR-193b-3p / hsa-miR-423-3p / hsa-miR-331-3p
HIV/HCV +	GATAD2B	0,028	14	hsa-miR-590-3p / hsa-miR-32-5p / hsa-miR-30a-5p / hsa-miR-505-5p / hsa-miR-193b-3p / hsa-miR-331-3p / hsa-miR-1260b / hsa-miR-642a-5p / hsa-miR-7-5p / hsa-miR-29a-5p / hsa-miR-589-3p / hsa-miR-1260a / hsa-miR-532-3p / hsa-miR-150-5p
HIV/HCV +	DUS1L	0,028	3	hsa-miR-7-5p / hsa-miR-24-3p / hsa-miR-193b-3p
HIV/HCV +	S100A8	0,028	3	hsa-miR-98-5p / hsa-miR-125b-5p / hsa-miR-24-3p
HIV/HCV +	CSTB	0,028	3	hsa-miR-877-5p / hsa-miR-423-5p / hsa-miR-125b-2-3p
HIV/HCV +	RHOV	0,029	5	hsa-miR-148b-3p / hsa-miR-9-5p / hsa-miR-193b-3p / hsa-miR-331-3p / hsa-miR-125b-5p
HIV/HCV +	PPP1R12C	0,032	6	hsa-miR-32-5p / hsa-miR-125b-2-3p / hsa-miR-1260b / hsa-miR-1260a / hsa-miR-532-3p / hsa-miR-1306-5p
HIV/HCV +	HUWE1	0,033	9	hsa-miR-34a-5p / hsa-miR-877-5p / hsa-miR-193b-3p / hsa-miR-29c-3p / hsa-miR-125b-5p / hsa-miR-196a-5p / hsa-miR-410-3p / hsa-miR-590-3p / hsa-miR-1277-5p
HIV/HCV +	DYRK2	0,034	17	hsa-miR-98-5p / hsa-miR-197-3p / hsa-miR-196a-5p / hsa-miR-24-3p / hsa-miR-590-3p / hsa-miR-193a-3p / hsa-miR-193b-3p / hsa-miR-3609 / hsa-miR-496 / hsa-miR-5701 / hsa-miR-1275 / hsa-miR-629-5p / hsa-miR-362-3p / hsa-miR-329-3p / hsa-miR-125a-5p / hsa-miR-125b-5p / hsa-miR-582-5p
HIV/HCV +	SMO	0,037	4	hsa-miR-125b-5p / hsa-miR-324-5p / hsa-miR-331-3p / hsa-miR-20a-3p
HIV/HCV +	IRAK1	0,037	6	hsa-miR-146a-5p / hsa-miR-146b-5p / hsa-miR-193b-3p / hsa-miR-423-3p / hsa-miR-328-3p / hsa-miR-142-3p
HIV/HCV +	PROSER1	0,037	4	hsa-miR-34a-5p / hsa-miR-98-5p / hsa-miR-24-3p / hsa-miR-196a-5p

HIV/HCV +	CASP9	0,037	4	hsa-miR-193b-3p / hsa-miR-7-5p / hsa-miR-582-5p / hsa-miR-34a-5p
HIV/HCV +	IL1RL2	0,037	5	hsa-miR-146a-5p / hsa-miR-146b-5p / hsa-miR-6511b-3p / hsa-miR-6511a-3p / hsa-miR-361-3p
HIV/HCV +	WDFY2	0,038	7	hsa-miR-30a-5p / hsa-miR-1260b / hsa-miR-1260a / hsa-miR-5100 / hsa-miR-532-3p / hsa-miR-150-5p / hsa-miR-24-3p
HIV/HCV +	NOTCH1	0,043	9	hsa-miR-24-3p / hsa-miR-34a-5p / hsa-miR-30a-5p / hsa-miR-139-5p / hsa-miR-204-5p / hsa-miR-9-5p / hsa-miR-410-3p / hsa-miR-146a-5p / hsa-miR-296-5p
HIV/HCV +	ABCC1	0,043	7	hsa-miR-7-5p / hsa-miR-134-5p / hsa-miR-9-5p / hsa-miR-98-5p / hsa-miR-125b-5p / hsa-miR-1291 / hsa-miR-361-3p
HIV/HCV +	VEGFA	0,043	17	hsa-miR-134-5p / hsa-miR-150-5p / hsa-miR-125a-5p / hsa-miR-34a-5p / hsa-miR-29c-3p / hsa-miR-9-5p / hsa-miR-1277-5p / hsa-miR-423-3p / hsa-miR-299-3p / hsa-miR-495-3p / hsa-miR-410-3p / hsa-miR-362-3p / hsa-miR-329-3p / hsa-miR-5193 / hsa-miR-126-5p / hsa-miR-101-5p / hsa-miR-296-5p
HIV/HCV +	PA2G4	0,043	6	hsa-miR-146a-5p / hsa-miR-24-3p / hsa-miR-423-5p / hsa-miR-324-5p / hsa-miR-7-5p / hsa-miR-423-3p
HIV/HCV +	GORASP2	0,043	4	hsa-miR-197-3p / hsa-miR-34a-5p / hsa-miR-877-5p / hsa-miR-24-3p
HIV/HCV +	ICAM1	0,043	8	hsa-miR-31-5p / hsa-miR-98-5p / hsa-miR-146a-5p / hsa-miR-485-5p / hsa-miR-2467-5p / hsa-miR-362-3p / hsa-miR-329-3p / hsa-miR-32-5p
HIV/HCV +	EZH2	0,043	9	hsa-miR-98-5p / hsa-miR-138-5p / hsa-miR-193b-3p / hsa-miR-1260b / hsa-miR-1260a / hsa-miR-532-3p / hsa-miR-150-5p / hsa-miR-320c / hsa-miR-101-5p
HIV/HCV +	SYNE2	0,043	5	hsa-miR-193b-3p / hsa-miR-148b-3p / hsa-miR-9-5p / hsa-miR-34a-5p / hsa-miR-485-5p
HIV/HCV +	SHCBP1	0,043	7	hsa-miR-193b-3p / hsa-miR-33a-5p / hsa-miR-32-5p / hsa-miR-146b-5p / hsa-miR-146a-5p / hsa-miR-589-5p / hsa-miR-3614-5p
HIV/HCV +	UGT8	0,043	8	hsa-miR-148b-3p / hsa-miR-1260b / hsa-miR-362-3p / hsa-miR-329-3p / hsa-miR-126-5p / hsa-miR-324-5p / hsa-miR-20a-3p / hsa-miR-4454
HIV/HCV +	OSBP2	0,043	4	hsa-miR-148b-3p / hsa-miR-122-5p / hsa-miR-1277-5p / hsa-miR-410-3p
HIV/HCV +	F2	0,043	11	hsa-miR-9-5p / hsa-miR-1260b / hsa-miR-1260a / hsa-miR-423-3p / hsa-miR-532-3p / hsa-miR-1343-3p / hsa-miR-1306-5p / hsa-miR-3614-5p / hsa-miR-122-5p / hsa-miR-25-5p / hsa-miR-485-5p
HIV/HCV +	SLC52A2	0,043	3	hsa-miR-122-5p / hsa-miR-7-5p / hsa-miR-24-3p
HIV/HCV +	UBE2L3	0,043	3	hsa-miR-122-5p / hsa-miR-423-5p / hsa-miR-125a-5p
HIV/HCV +	ANXA7	0,043	3	hsa-miR-122-5p / hsa-miR-324-5p / hsa-miR-196a-5p

HIV/HCV +	PHPT1	0,043	3	hsa-miR-122-5p / hsa-miR-877-5p / hsa-miR-197-3p
HIV/HCV +	SRRM2	0,043	6	hsa-miR-34a-5p / hsa-miR-877-5p / hsa-miR-505-5p / hsa-miR-331-3p / hsa-miR-125b-5p / hsa-miR-31-5p
HIV/HCV +	TYMS	0,043	5	hsa-miR-193b-3p / hsa-miR-34a-5p / hsa-miR-99a-5p / hsa-miR-196a-5p / hsa-miR-193a-3p
HIV/HCV +	LAMC2	0,043	4	hsa-miR-148b-3p / hsa-miR-7-5p / hsa-miR-29c-3p / hsa-miR-146a-5p
HIV/HCV +	SERTAD2	0,043	11	hsa-miR-7-5p / hsa-miR-98-5p / hsa-miR-33a-5p / hsa-miR-324-5p / hsa-miR-1277-5p / hsa-miR-4531 / hsa-miR-146b-5p / hsa-miR-146a-5p / hsa-miR-32-5p / hsa-miR-134-5p / hsa-miR-31-5p
HIV/HCV +	B4GALT3	0,043	3	hsa-miR-34a-5p / hsa-miR-98-5p / hsa-miR-125b-5p
HIV/HCV +	RHOBTB3	0,043	11	hsa-miR-98-5p / hsa-miR-432-5p / hsa-miR-379-5p / hsa-miR-146b-5p / hsa-miR-146a-5p / hsa-miR-664b-5p / hsa-miR-381-3p / hsa-miR-181b-2-3p / hsa-miR-411-5p / hsa-miR-590-3p / hsa-miR-342-5p
HIV/HCV +	MIS18A	0,043	6	hsa-miR-24-3p / hsa-miR-505-5p / hsa-miR-1260b / hsa-miR-1260a / hsa-miR-532-3p / hsa-miR-150-5p
HIV/HCV +	POLR3H	0,043	4	hsa-miR-423-5p / hsa-miR-423-3p / hsa-miR-324-5p / hsa-miR-331-3p
HIV/HCV +	TRAP1	0,043	6	hsa-miR-361-3p / hsa-miR-193b-3p / hsa-miR-324-5p / hsa-miR-331-3p / hsa-miR-197-3p / hsa-miR-196a-5p
HIV/HCV +	COX7C	0,043	3	hsa-miR-193b-3p / hsa-miR-331-3p / hsa-miR-125b-5p
HIV/HCV +	SPEN	0,043	7	hsa-miR-193b-3p / hsa-miR-423-3p / hsa-miR-324-5p / hsa-miR-331-3p / hsa-miR-328-3p / hsa-miR-125b-5p / hsa-miR-196a-5p
HIV/HCV +	GGT7	0,043	4	hsa-miR-331-3p / hsa-miR-197-3p / hsa-miR-6511b-3p / hsa-miR-6511a-3p
HIV/HCV +	SIRT7	0,043	3	hsa-miR-34a-5p / hsa-miR-125b-5p / hsa-miR-125a-5p
HIV/HCV +	UHRF1	0,045	5	hsa-miR-146a-5p / hsa-miR-34a-5p / hsa-miR-7-5p / hsa-miR-146b-5p / hsa-miR-9-5p
HIV/HCV-	BCL2	0	18	hsa-miR-204-5p / hsa-miR-29c-3p / hsa-miR-181c-5p / hsa-miR-33b-5p / hsa-miR-451a / hsa-miR-125b-5p / hsa-miR-200b-3p / hsa-miR-7-5p / hsa-miR-148a-3p / hsa-miR-182-5p / hsa-miR-143-3p / hsa-miR-126-3p / hsa-miR-18a-5p / hsa-miR-125a-5p / hsa-miR-224-5p / hsa-miR-139-5p / hsa-miR-376c-3p / hsa-miR-98-5p
HIV/HCV-	MEGF9	0,008	6	hsa-miR-151a-3p / hsa-miR-877-5p / hsa-miR-197-3p / hsa-miR-125b-5p / hsa-miR-125a-5p / hsa-miR-432-5p
HIV/HCV-	NFKB2	0,018	4	hsa-miR-193b-3p / hsa-miR-98-5p / hsa-miR-423-5p / hsa-miR-143-3p
HIV/HCV-	PRKAB1	0,018	9	hsa-miR-122-5p / hsa-miR-148b-3p / hsa-miR-642a-5p / hsa-miR-627-3p / hsa-miR-432-5p / hsa-miR-1260b / hsa-

				miR-1260a / hsa-miR-532-3p / hsa-miR-150-5p
HIV/HCV-	MTRNR2L2	0,018	5	hsa-miR-376c-3p / hsa-miR-381-3p / hsa-miR-485-3p / hsa-miR-493-5p / hsa-miR-382-5p
HIV/HCV-	BCL11A	0,027	7	hsa-miR-148b-3p / hsa-miR-29c-3p / hsa-miR-196a-5p / hsa-miR-32-5p / hsa-miR-30a-5p / hsa-miR-2110 / hsa-miR-376c-3p
HIV/HCV-	HK2	0,029	5	hsa-miR-143-3p / hsa-miR-125a-5p / hsa-miR-125b-5p / hsa-miR-98-5p / hsa-miR-199a-5p
HIV/HCV-	ERO1B	0,029	5	hsa-miR-33a-5p / hsa-miR-1185-1-3p / hsa-miR-376c-3p / hsa-miR-432-5p / hsa-miR-376a-3p
HIV/HCV-	MDN1	0,029	7	hsa-miR-642a-5p / hsa-miR-1260b / hsa-miR-146b-5p / hsa-miR-146a-5p / hsa-miR-589-5p / hsa-miR-362-3p / hsa-miR-329-3p
HIV/HCV-	TMEM170B	0,029	12	hsa-miR-1271-5p / hsa-miR-376c-3p / hsa-miR-495-3p / hsa-miR-182-5p / hsa-miR-183-5p / hsa-miR-33b-5p / hsa-miR-33a-5p / hsa-miR-877-5p / hsa-miR-4685-3p / hsa-miR-1248 / hsa-miR-3916 / hsa-miR-7977
HIV/HCV-	PIK3CG	0,029	10	hsa-miR-126-3p / hsa-miR-148b-3p / hsa-miR-7-5p / hsa-miR-142-3p / hsa-miR-362-3p / hsa-miR-329-3p / hsa-miR-410-3p / hsa-miR-548e-3p / hsa-miR-127-3p / hsa-miR-125a-5p
HIV/HCV-	PRKCH	0,029	7	hsa-miR-376c-3p / hsa-miR-432-5p / hsa-miR-376a-3p / hsa-miR-493-3p / hsa-miR-1343-3p / hsa-miR-26a-2-3p / hsa-miR-24-3p
HIV/HCV-	COPA	0,029	9	hsa-miR-193b-3p / hsa-miR-1260b / hsa-miR-127-5p / hsa-miR-376a-3p / hsa-miR-142-3p / hsa-miR-146b-5p / hsa-miR-146a-5p / hsa-miR-589-5p / hsa-miR-122-5p
HIV/HCV-	BBS4	0,029	4	hsa-miR-376c-3p / hsa-miR-127-5p / hsa-miR-432-5p / hsa-miR-382-5p
HIV/HCV-	IKBKB	0,044	5	hsa-miR-199a-5p / hsa-miR-151a-3p / hsa-miR-451a / hsa-miR-148a-3p / hsa-miR-200b-3p
HIV/HCV-	SHCBP1	0,044	8	hsa-miR-193b-3p / hsa-miR-33a-5p / hsa-miR-32-5p / hsa-miR-200b-3p / hsa-miR-146b-5p / hsa-miR-146a-5p / hsa-miR-589-5p / hsa-miR-3614-5p
HIV/HCV-	TLR2	0,044	5	hsa-miR-146a-5p / hsa-miR-154-5p / hsa-miR-101-5p / hsa-miR-143-3p / hsa-miR-23a-5p
HIV/HCV-	CXCR4	0,045	6	hsa-miR-146a-5p / hsa-miR-224-5p / hsa-miR-150-5p / hsa-miR-139-5p / hsa-miR-126-3p / hsa-miR-204-5p
HIV/HCV-	KRAS	0,045	14	hsa-miR-143-3p / hsa-miR-181c-5p / hsa-miR-126-3p / hsa-miR-193b-3p / hsa-miR-134-5p / hsa-miR-224-5p / hsa-miR-452-5p / hsa-miR-3916 / hsa-miR-200b-3p / hsa-miR-193a-3p / hsa-miR-183-5p / hsa-miR-877-5p / hsa-miR-642a-5p / hsa-miR-199a-5p
HIV/HCV-	SERPINE1	0,045	7	hsa-miR-204-5p / hsa-miR-99a-5p / hsa-miR-143-3p / hsa-miR-148a-3p / hsa-

				miR-486-5p / hsa-miR-30a-5p / hsa-miR-224-5p
HIV/HCV-	FBXO7	0,045	3	hsa-miR-122-5p / hsa-miR-877-5p / hsa-miR-127-5p
HIV/HCV-	S100A8	0,045	3	hsa-miR-98-5p / hsa-miR-125b-5p / hsa-miR-24-3p
HIV/HCV-	DUS1L	0,045	3	hsa-miR-7-5p / hsa-miR-24-3p / hsa-miR-193b-3p
HIV/HCV-	CSTB	0,045	3	hsa-miR-877-5p / hsa-miR-423-5p / hsa-miR-125b-2-3p
HIV/HCV-	HNRNPK	0,045	7	hsa-miR-450a-5p / hsa-miR-328-3p / hsa-miR-125b-5p / hsa-miR-1260b / hsa-miR-6511a-3p / hsa-miR-6511b-3p / hsa-miR-590-3p
HIV/HCV-	EZH2	0,046	10	hsa-miR-199a-5p / hsa-miR-98-5p / hsa-miR-193b-3p / hsa-miR-200b-3p / hsa-miR-1260b / hsa-miR-1260a / hsa-miR-532-3p / hsa-miR-150-5p / hsa-miR-126-3p / hsa-miR-101-5p
HIV/HCV-	WNT1	0,048	5	hsa-miR-122-5p / hsa-miR-200b-3p / hsa-miR-148a-3p / hsa-miR-148b-3p / hsa-miR-139-5p
HIV	BCL2	0,0059303 6	15	hsa-miR-204-5p / hsa-miR-29c-3p / hsa-miR-181c-5p / hsa-miR-33b-5p / hsa-miR-125b-5p / hsa-miR-7-5p / hsa-miR-148a-3p / hsa-miR-182-5p / hsa-miR-143-3p / hsa-miR-126-3p / hsa-miR-125a-5p / hsa-miR-224-5p / hsa-miR-494-3p / hsa-miR-139-5p / hsa-miR-98-5p
HIV	MEGF9	0,0059303 6	6	hsa-miR-151a-3p / hsa-miR-877-5p / hsa-miR-197-3p / hsa-miR-125b-5p / hsa-miR-125a-5p / hsa-miR-432-5p
HIV	CXCR4	0,0109388 5	7	hsa-miR-146a-5p / hsa-miR-224-5p / hsa-miR-150-5p / hsa-miR-139-5p / hsa-miR-126-3p / hsa-miR-494-3p / hsa-miR-204-5p
HIV	EZH2	0,0109388 5	11	hsa-miR-199a-5p / hsa-miR-98-5p / hsa-miR-138-5p / hsa-miR-193b-3p / hsa-miR-1260b / hsa-miR-1260a / hsa-miR-3150b-3p / hsa-miR-532-3p / hsa-miR-150-5p / hsa-miR-126-3p / hsa-miR-101-5p
HIV	SERPINE1	0,0109388 5	8	hsa-miR-204-5p / hsa-miR-99a-5p / hsa-miR-143-3p / hsa-miR-138-5p / hsa-miR-148a-3p / hsa-miR-486-5p / hsa-miR-30a-5p / hsa-miR-224-5p
HIV	NFKB2	0,0109388 5	4	hsa-miR-193b-3p / hsa-miR-98-5p / hsa-miR-423-5p / hsa-miR-143-3p
HIV	BCL11A	0,0109388 5	7	hsa-miR-148b-3p / hsa-miR-29c-3p / hsa-miR-196a-5p / hsa-miR-32-5p / hsa-miR-30a-5p / hsa-miR-2110 / hsa-miR-138-5p
HIV	SLC45A3	0,0235680 2	4	hsa-miR-138-5p / hsa-miR-126-3p / hsa-miR-126-5p / hsa-miR-32-5p
HIV	HK2	0,0251052 5	5	hsa-miR-143-3p / hsa-miR-125a-5p / hsa-miR-125b-5p / hsa-miR-98-5p / hsa-miR-199a-5p
HIV	TLR2	0,0392282 8	5	hsa-miR-146a-5p / hsa-miR-154-5p / hsa-miR-101-5p / hsa-miR-143-3p / hsa-miR-23a-5p
HIV	AKT1	0,0392282 8	13	hsa-miR-125b-5p / hsa-miR-143-3p / hsa-miR-193b-3p / hsa-miR-542-3p / hsa-miR-27a-5p / hsa-miR-496 / hsa-

				miR-99a-5p / hsa-miR-138-5p / hsa-miR-409-3p / hsa-miR-125a-5p / hsa-miR-494-3p / hsa-miR-185-3p / hsa-miR-126-3p
HIV	FBXO7	0,04772666	3	hsa-miR-122-5p / hsa-miR-877-5p / hsa-miR-127-5p
HIV	S100A8	0,04772666	3	hsa-miR-98-5p / hsa-miR-125b-5p / hsa-miR-24-3p
HIV	RHOBTB3	0,04772666	12	hsa-miR-98-5p / hsa-miR-432-5p / hsa-miR-379-5p / hsa-miR-146b-5p / hsa-miR-146a-5p / hsa-miR-381-3p / hsa-miR-4435 / hsa-miR-181b-2-3p / hsa-miR-411-5p / hsa-miR-452-5p / hsa-miR-590-3p / hsa-miR-342-5p
HIV	DUS1L	0,04772666	3	hsa-miR-7-5p / hsa-miR-24-3p / hsa-miR-193b-3p

Table S3. Pathway enrichment analysis. Enriched target genes in TS4 were used to identify potentially altered pathways. This table indicates the enriched KEGG pathways for significant miRNA-targets (see ST4) in each analysis (HIV/HCV+ vs HC, HIV/HCV- vs HC, and HIV vs HC).

Analysis	KEGG Name	Term ID	FDR	miRNA-targets
HIV/HCV+	Toxoplasmosis	KEGG:05145	0	LAMC2 / PIK3CG / NFKB1 / CASP9 / TLR2 / IRAK1
HIV/HCV+	Legionellosis	KEGG:05134	0,007	NFKB2 / NFKB1 / CASP9 / TLR2
HIV/HCV+	Epstein-Barr virus infection	KEGG:05169	0,012	NFKB2 / ICAM1 / NFKB1 / CASP9 / TLR2 / IRAK1
HIV/HCV+	Antifolate resistance	KEGG:01523	0,021	ABCC1 / NFKB1 / TYMS
HIV/HCV+	PI3K-Akt signaling pathway	KEGG:04151	0,043	LAMC2 / PIK3CG / NFKB1 / VEGFA / CASP9 / TLR2 / MCL1
HIV/HCV-	Human immunodeficiency virus 1 infection	KEGG:05170	0,003	IKBKB / CXCR4 / KRAS / TLR2 / BCL2
HIV/HCV-	Toxoplasmosis	KEGG:05145	0,004	IKBKB / PIK3CG / TLR2 / BCL2
HIV/HCV-	Apelin signaling pathway	KEGG:04371	0,01	PIK3CG / SERPINE1 / PRKAB1 / KRAS
HIV/HCV-	Insulin signaling pathway	KEGG:04910	0,01	IKBKB / PRKAB1 / KRAS / HK2
HIV/HCV-	Hepatitis B	KEGG:05161	0,019	IKBKB / KRAS / TLR2 / BCL2
HIV/HCV-	MicroRNAs in cancer	KEGG:05206	0,021	IKBKB / EZH2 / KRAS / HNRNPK / BCL2
HIV/HCV-	Pathways in cancer	KEGG:05200	0,032	NFKB2 / IKBKB / CXCR4 / WNT1 / KRAS / BCL2
HIV/HCV-	Chemokine signaling pathway	KEGG:04062	0,034	IKBKB / PIK3CG / CXCR4 / KRAS
HIV/HCV-	PI3K-Akt signaling pathway	KEGG:04151	0,039	IKBKB / PIK3CG / KRAS / TLR2 / BCL2
HIV/HCV-	Epstein-Barr virus infection	KEGG:05169	0,041	NFKB2 / IKBKB / TLR2 / BCL2
HIV/HCV-	PD-L1 expression and PD-1 checkpoint pathway in cancer	KEGG:05235	0,048	IKBKB / KRAS / TLR2
HIV	HIF-1 signaling pathway	KEGG:04066	0	SERPINE1 / AKT1 / HK2 / BCL2
HIV	Epstein-Barr virus infection	KEGG:05169	0,004	NFKB2 / TLR2 / AKT1 / BCL2
HIV	Human immunodeficiency virus 1 infection	KEGG:05170	0,005	CXCR4 / TLR2 / AKT1 / BCL2

HIV	AGE-RAGE signaling pathway in diabetic complications	KEGG:04933	0,013	SERPINE1 / AKT1 / BCL2
HIV	Chagas disease	KEGG:05142	0,013	SERPINE1 / TLR2 / AKT1
HIV	Toxoplasmosis	KEGG:05145	0,017	TLR2 / AKT1 / BCL2
HIV	Measles	KEGG:05162	0,033	TLR2 / AKT1 / BCL2

Table S4. Differentially expressed miRNAs in HIV/HCV- patients. Significant differentially expressed (SDE) miRNAs in HIV/HCV- patients compared to healthy controls. Each column represent: the SDE miRNA identifiers (miRBase format), the Log2FC indicating the expression fold change between HIV/HCV- and healthy patients, and the adjusted p-value (FDR, corrected for multiple testing with the Benjamin–Hochberg procedure).

miRNAs	FC	FDR
hsa-miR-101-5p	0,542	0
hsa-miR-10395-3p	0,432	0
hsa-miR-10401-3p	0,286	0
hsa-miR-11400	2,15	0
hsa-miR-1180-3p	0,657	0,004
hsa-miR-1185-1-3p	1,541	0,04
hsa-miR-12135	0,475	0
hsa-miR-122-5p	0,37	0
hsa-miR-1246	0,111	0
hsa-miR-1248	0,091	0
hsa-miR-1249-3p	0,258	0
hsa-miR-125a-5p	0,451	0
hsa-miR-125b-2-3p	0,64	0,004
hsa-miR-125b-5p	0,331	0
hsa-miR-126-3p	1,736	0
hsa-miR-126-5p	2,119	0
hsa-miR-1260a	0,461	0
hsa-miR-1260b	0,47	0
hsa-miR-1268a	0,565	0
hsa-miR-1268b	0,616	0,001
hsa-miR-127-3p	2,135	0
hsa-miR-127-5p	1,811	0,005
hsa-miR-1271-5p	0,634	0,001
hsa-miR-1285-3p	0,564	0

hsa-miR-1291	0,449	0
hsa-miR-1304-3p	1,642	0
hsa-miR-1306-5p	0,404	0
hsa-miR-1307-5p	0,549	0,006
hsa-miR-132-5p	0,512	0
hsa-miR-134-5p	2,537	0
hsa-miR-1343-3p	0,483	0
hsa-miR-136-3p	1,739	0,008
hsa-miR-139-5p	2,434	0
hsa-miR-142-3p	0,53	0
hsa-miR-143-3p	2,422	0
hsa-miR-146a-5p	2,811	0
hsa-miR-146b-5p	2,314	0
hsa-miR-148a-3p	1,526	0
hsa-miR-148a-5p	2,22	0
hsa-miR-148b-3p	1,792	0
hsa-miR-148b-5p	1,636	0
hsa-miR-150-5p	0,42	0
hsa-miR-151a-3p	1,832	0
hsa-miR-1538	0,637	0,005
hsa-miR-154-5p	2,316	0
hsa-miR-181b-2-3p	0,495	0
hsa-miR-181c-3p	1,58	0
hsa-miR-181c-5p	1,564	0
hsa-miR-182-5p	1,653	0,002
hsa-miR-183-5p	1,892	0,025
hsa-miR-185-3p	1,665	0
hsa-miR-18a-5p	1,667	0
hsa-miR-191-5p	1,535	0
hsa-miR-193a-3p	0,408	0
hsa-miR-193b-3p	0,506	0
hsa-miR-196a-5p	1,835	0,004
hsa-miR-197-3p	0,497	0
hsa-miR-199a-5p	1,937	0
hsa-miR-200b-3p	0,628	0
hsa-miR-204-5p	1,836	0,004
hsa-miR-21-3p	1,894	0
hsa-miR-210-5p	0,32	0
hsa-miR-2110	0,614	0
hsa-miR-224-5p	1,65	0,01
hsa-miR-23a-5p	0,286	0
hsa-miR-24-3p	1,609	0
hsa-miR-2467-5p	1,777	0
hsa-miR-25-5p	0,501	0
hsa-miR-26a-2-3p	0,64	0,007
hsa-miR-27a-5p	0,616	0,001

hsa-miR-296-5p	0,344	0
hsa-miR-29a-5p	0,622	0
hsa-miR-29c-3p	0,575	0
hsa-miR-30a-5p	1,905	0
hsa-miR-31-5p	0,65	0,002
hsa-miR-3120-3p	1,564	0,005
hsa-miR-3142	0,362	0
hsa-miR-32-3p	0,53	0
hsa-miR-32-5p	0,5	0
hsa-miR-324-5p	1,639	0
hsa-miR-328-3p	0,614	0
hsa-miR-329-3p	1,941	0,003
hsa-miR-337-3p	1,529	0,049
hsa-miR-338-3p	0,597	0
hsa-miR-33a-5p	0,634	0,003
hsa-miR-33b-5p	0,633	0,003
hsa-miR-342-5p	0,545	0
hsa-miR-3605-3p	0,44	0
hsa-miR-3609	0,253	0
hsa-miR-3614-5p	1,544	0,024
hsa-miR-3615	0,649	0
hsa-miR-362-3p	0,598	0,001
hsa-miR-362-5p	3,003	0
hsa-miR-3651	0,483	0
hsa-miR-370-3p	1,77	0,006
hsa-miR-376a-3p	2,037	0,002
hsa-miR-376c-3p	2,564	0
hsa-miR-377-5p	1,999	0
hsa-miR-379-5p	2,844	0
hsa-miR-381-3p	2,15	0
hsa-miR-382-5p	1,882	0,001
hsa-miR-3916	0,608	0,001
hsa-miR-3960	0,185	0
hsa-miR-409-3p	1,755	0,001
hsa-miR-410-3p	2,28	0
hsa-miR-411-3p	1,693	0,008
hsa-miR-411-5p	2,276	0
hsa-miR-423-5p	0,475	0
hsa-miR-4286	0,62	0
hsa-miR-431-3p	1,559	0,024
hsa-miR-431-5p	1,6	0,011
hsa-miR-432-5p	1,999	0
hsa-miR-4433b-3p	0,589	0,024
hsa-miR-4433b-5p	0,643	0,03
hsa-miR-4454	0,416	0
hsa-miR-4508	0,223	0

hsa-miR-450a-5p	0,631	0,001
hsa-miR-451a	2,696	0,002
hsa-miR-452-5p	2,467	0
hsa-miR-4645-3p	1,98	0
hsa-miR-4685-3p	0,543	0
hsa-miR-4687-5p	0,326	0
hsa-miR-4772-3p	0,397	0
hsa-miR-4772-5p	0,422	0
hsa-miR-485-3p	1,533	0,033
hsa-miR-486-5p	1,671	0
hsa-miR-493-3p	1,892	0
hsa-miR-493-5p	1,878	0,001
hsa-miR-495-3p	2,031	0,002
hsa-miR-502-3p	0,576	0
hsa-miR-505-5p	0,404	0
hsa-miR-5100	0,498	0
hsa-miR-532-3p	0,544	0
hsa-miR-548ad-5p	0,634	0
hsa-miR-548ae-5p	0,624	0,001
hsa-miR-548ag	1,736	0,001
hsa-miR-548au-5p	0,598	0
hsa-miR-548ay-5p	0,608	0
hsa-miR-548e-3p	0,661	0
hsa-miR-5701	0,439	0
hsa-miR-582-3p	1,657	0
hsa-miR-584-5p	1,683	0
hsa-miR-589-5p	1,563	0
hsa-miR-590-3p	0,527	0
hsa-miR-624-5p	0,664	0,007
hsa-miR-627-3p	0,656	0,007
hsa-miR-629-3p	0,591	0
hsa-miR-629-5p	1,908	0
hsa-miR-642a-3p	0,534	0
hsa-miR-642a-5p	0,578	0
hsa-miR-6511a-3p	0,585	0
hsa-miR-6511b-3p	0,45	0
hsa-miR-654-3p	1,518	0,023
hsa-miR-654-5p	2,138	0
hsa-miR-6724-5p	0,622	0,001
hsa-miR-6803-3p	0,306	0
hsa-miR-6837-3p	2,009	0
hsa-miR-7-5p	2,177	0
hsa-miR-7704	0,405	0
hsa-miR-7849-3p	1,719	0
hsa-miR-7976	0,569	0
hsa-miR-7977	0,366	0

hsa-miR-877-5p	0,447	0
hsa-miR-889-3p	1,822	0,003
hsa-miR-95-3p	0,647	0
hsa-miR-98-5p	1,588	0
hsa-miR-9985	0,438	0
hsa-miR-99a-5p	0,46	0
hsa-miR-99b-3p	1,531	0

Table S5. Differentially expressed miRNAs in HIV patients. Significant differentially expressed (SDE) miRNAs in HIV patients compared to healthy controls. Each column represent: the SDE miRNA identifiers (miRBase format), the Log2FC indicating the expression fold change between HIV and healthy patients, and the adjusted p-value (FDR, corrected for multiple testing with the Benjamin–Hochberg procedure).

miRNAs	LogFC	FDR
hsa-miR-101-5p	-0,837	0
hsa-miR-10395-3p	-0,944	0
hsa-miR-10399-3p	0,655	0
hsa-miR-10401-3p	-1,85	0
hsa-miR-11400	1,088	0
hsa-miR-12135	-1,236	0
hsa-miR-122-5p	-1,345	0
hsa-miR-1246	-3,446	0
hsa-miR-1248	-2,812	0
hsa-miR-1249-3p	-1,626	0
hsa-miR-125a-5p	-1,014	0
hsa-miR-125b-5p	-1,161	0
hsa-miR-126-3p	0,613	0,001
hsa-miR-126-5p	0,887	0
hsa-miR-1260a	-1,239	0
hsa-miR-1260b	-1,218	0
hsa-miR-1268a	-0,645	0,001
hsa-miR-127-3p	1,132	0
hsa-miR-127-5p	0,68	0,032
hsa-miR-1277-5p	-0,713	0,002
hsa-miR-1285-3p	-0,904	0
hsa-miR-1291	-0,969	0
hsa-miR-1306-5p	-1,1	0
hsa-miR-1307-5p	-1,023	0
hsa-miR-132-5p	-0,864	0
hsa-miR-134-5p	1,144	0

hsa-miR-1343-3p	-0,738	0,001
hsa-miR-138-5p	-0,643	0,003
hsa-miR-139-5p	1,109	0
hsa-miR-142-3p	-0,943	0
hsa-miR-143-3p	1,247	0
hsa-miR-144-5p	0,786	0,043
hsa-miR-146a-5p	1,545	0
hsa-miR-146b-5p	1,187	0
hsa-miR-148a-3p	0,665	0
hsa-miR-148a-5p	1,259	0
hsa-miR-148b-3p	0,94	0
hsa-miR-148b-5p	0,732	0
hsa-miR-150-5p	-1,164	0
hsa-miR-151a-3p	0,954	0
hsa-miR-152-5p	0,897	0
hsa-miR-154-5p	1,075	0
hsa-miR-181b-2-3p	-0,907	0
hsa-miR-181c-3p	0,692	0
hsa-miR-181c-5p	0,658	0
hsa-miR-182-5p	0,886	0
hsa-miR-1843	0,64	0
hsa-miR-185-3p	0,824	0
hsa-miR-191-5p	0,768	0
hsa-miR-193a-3p	-1,167	0
hsa-miR-193b-3p	-0,757	0
hsa-miR-196a-5p	0,99	0,001
hsa-miR-197-3p	-0,677	0
hsa-miR-199a-5p	0,808	0
hsa-miR-204-5p	0,88	0,004
hsa-miR-20a-3p	-0,821	0
hsa-miR-21-3p	1,089	0
hsa-miR-210-5p	-1,33	0
hsa-miR-2110	-0,629	0
hsa-miR-223-5p	0,669	0
hsa-miR-224-5p	0,602	0,031
hsa-miR-23a-5p	-1,651	0
hsa-miR-24-3p	0,611	0
hsa-miR-2467-5p	0,82	0
hsa-miR-25-5p	-0,744	0
hsa-miR-26a-2-3p	-0,757	0
hsa-miR-27a-5p	-0,691	0
hsa-miR-296-5p	-1,605	0
hsa-miR-29a-5p	-0,954	0
hsa-miR-29c-3p	-0,821	0
hsa-miR-30a-5p	1,017	0
hsa-miR-31-5p	-0,655	0

hsa-miR-3142	-1,302	0
hsa-miR-3150b-3p	0,612	0
hsa-miR-3176	0,662	0
hsa-miR-32-3p	-0,873	0
hsa-miR-32-5p	-1,039	0
hsa-miR-324-5p	0,786	0
hsa-miR-338-3p	-0,803	0
hsa-miR-33a-5p	-0,939	0
hsa-miR-33b-5p	-0,849	0
hsa-miR-342-5p	-0,75	0
hsa-miR-3605-3p	-0,912	0
hsa-miR-3609	-1,733	0
hsa-miR-3613-5p	-0,59	0,005
hsa-miR-3614-5p	0,728	0,002
hsa-miR-362-3p	-0,993	0
hsa-miR-362-5p	1,424	0
hsa-miR-3651	-0,854	0,001
hsa-miR-370-3p	0,718	0,021
hsa-miR-377-5p	0,722	0,008
hsa-miR-379-5p	1,266	0
hsa-miR-381-3p	0,948	0
hsa-miR-382-5p	0,787	0,002
hsa-miR-3960	-2,118	0
hsa-miR-409-3p	0,752	0,005
hsa-miR-410-3p	1,005	0
hsa-miR-411-5p	1,045	0
hsa-miR-423-5p	-0,859	0
hsa-miR-425-3p	0,627	0
hsa-miR-432-5p	0,904	0,002
hsa-miR-4433b-3p	-0,726	0,049
hsa-miR-4433b-5p	-0,973	0
hsa-miR-4435	0,788	0,002
hsa-miR-4454	-1,087	0
hsa-miR-4508	-2,176	0
hsa-miR-450a-5p	-0,614	0,002
hsa-miR-4516	-1,554	0
hsa-miR-452-5p	0,951	0,003
hsa-miR-4645-3p	1,136	0
hsa-miR-4687-5p	-1,316	0
hsa-miR-4746-5p	0,793	0
hsa-miR-4772-3p	-1,333	0
hsa-miR-4772-5p	-1,409	0
hsa-miR-486-5p	0,919	0
hsa-miR-493-3p	0,921	0,001
hsa-miR-493-5p	0,751	0,007
hsa-miR-494-3p	-0,732	0,014

hsa-miR-496	1,125	0
hsa-miR-502-3p	-0,662	0
hsa-miR-505-5p	-1,106	0
hsa-miR-5100	-0,654	0
hsa-miR-532-3p	-0,642	0
hsa-miR-542-3p	-0,623	0,019
hsa-miR-548ae-5p	-0,695	0
hsa-miR-548ag	0,888	0
hsa-miR-548au-5p	-0,713	0
hsa-miR-5701	-0,847	0
hsa-miR-582-3p	0,895	0
hsa-miR-584-5p	0,702	0,002
hsa-miR-589-5p	0,794	0
hsa-miR-590-3p	-1,105	0
hsa-miR-624-5p	-0,722	0,001
hsa-miR-629-5p	0,98	0
hsa-miR-642a-3p	-0,898	0
hsa-miR-642a-5p	-0,781	0
hsa-miR-6511a-3p	-0,622	0
hsa-miR-6511b-3p	-1,104	0
hsa-miR-654-5p	1,261	0
hsa-miR-6803-3p	-1,583	0
hsa-miR-6837-3p	1,12	0
hsa-miR-6852-5p	0,65	0
hsa-miR-7-5p	0,907	0
hsa-miR-769-5p	0,616	0
hsa-miR-7704	-1,136	0
hsa-miR-7849-3p	0,9	0
hsa-miR-7977	-1,248	0
hsa-miR-877-5p	-1,01	0
hsa-miR-889-3p	0,592	0,037
hsa-miR-98-5p	0,7	0
hsa-miR-9985	-1,144	0
hsa-miR-99a-5p	-0,783	0
hsa-miR-99b-3p	0,782	0

Table S6. Top enriched target genes for specific miRNA deregulation signature in HIV/HCV+ patients. Here we have included the target gene symbol (Gene_symbol), the adjusted p-value (FDR, corrected p-value for multiple testing performed with the Benjamin–Hochberg procedure), the number of SDE miRNAs that interact with each enriched gene (Num_interactions), the miRNA identifier in miRBase format and its deregulation sense (miRNA_expression).

Gene_symbol	FDR	Num_interactions	miRNAs_expression	miRNAs
ATG5	0,00758142	2	Up	hsa-miR-9-5p / hsa-miR-34a-5p
BIK	0,00758142	2	Up	hsa-miR-9-5p / hsa-miR-34a-5p
CASP9	0,00758142	2	Up	hsa-miR-582-5p / hsa-miR-34a-5p
DSP	0,00758142	2	Up	hsa-miR-9-5p / hsa-miR-34a-5p
ITGB3BP	0,00758142	2	Up	hsa-miR-9-5p / hsa-miR-299-3p
MYCN	0,00758142	3	Up	hsa-miR-34a-5p / hsa-miR-582-5p / hsa-miR-299-3p
PDGFRB	0,00810835	2	Up	hsa-miR-34a-5p / hsa-miR-9-5p
HIST1H3F	0,01009616	2	Up	hsa-miR-34a-5p / hsa-miR-9-5p
HIST1H4H	0,01009616	2	Up	hsa-miR-9-5p / hsa-miR-34a-5p
PRKAA1	0,01009616	3	Up	hsa-miR-582-5p / hsa-miR-299-3p / hsa-miR-9-5p
ABCA2	0,02038456	3	Down	hsa-miR-485-5p / hsa-miR-320d / hsa-miR-320c
SPTBN1	0,02038456	3	Down	hsa-miR-589-3p / hsa-miR-423-3p / hsa-miR-331-3p
ADCY1	0,02329885	2	Down	hsa-miR-423-3p / hsa-miR-331-3p
DDX56	0,02329885	2	Down	hsa-miR-423-3p / hsa-miR-331-3p
EIF2B5	0,02329885	3	Down	hsa-miR-361-3p / hsa-miR-320d / hsa-miR-320c
FLII	0,02329885	2	Down	hsa-miR-423-3p / hsa-miR-331-3p
POLR2L	0,02329885	2	Down	hsa-miR-423-3p / hsa-miR-331-3p
RPS2	0,02329885	3	Down	hsa-miR-361-3p / hsa-miR-423-3p / hsa-miR-331-3p
SYNGR1	0,02329885	4	Down	hsa-miR-3196 / hsa-miR-486-3p / hsa-miR-1908-5p / hsa-miR-5193
NACC1	0,02530053	7	Down	hsa-miR-1275 / hsa-miR-1908-5p / hsa-miR-331-3p / hsa-miR-3196 / hsa-miR-361-3p / hsa-miR-485-5p / hsa-miR-486-3p

Table S7. Top enriched target genes for specific miRNA deregulation signature in HIV/HCV- patients. Here we have included the target gene symbol (Gene_symbol), the adjusted p-value (FDR, corrected p-value for multiple testing performed with the Benjamin–Hochberg procedure), the number of SDE miRNAs that interact with each enriched gene (Num_interactions), the miRNA identifier in miRBase format and its deregulation sense (miRNA_expression).

Gene_symbol	FDR	Num_interactions	miRNAs_expression	miRNAs
PANK3	0,01212072	5	Up	hsa-miR-183-5p / hsa-miR-485-3p / hsa-miR-1185-1-3p / hsa-miR-1304-3p / hsa-miR-337-3p
BMI1	0,01760736	4	Up	hsa-miR-183-5p / hsa-miR-411-3p / hsa-miR-3120-3p / hsa-miR-376c-3p
CD300LB	0,01760736	2	Up	hsa-miR-431-5p / hsa-miR-654-3p
CLDN1	0,01760736	3	Up	hsa-miR-376c-3p / hsa-miR-337-3p / hsa-miR-1304-3p
MTRNR2L2	0,01760736	2	Up	hsa-miR-376c-3p / hsa-miR-485-3p
NXPE3	0,01760736	3	Up	hsa-miR-337-3p / hsa-miR-411-3p / hsa-miR-431-5p
ERO1B	0,01935422	2	Up	hsa-miR-1185-1-3p / hsa-miR-376c-3p
NBEA	0,0257402	2	Up	hsa-miR-18a-5p / hsa-miR-485-3p
GP2	0,0318774	3	Up	hsa-miR-183-5p / hsa-miR-1304-3p / hsa-miR-431-5p
CRIM1	0,03275743	3	Up	hsa-miR-18a-5p / hsa-miR-337-3p / hsa-miR-183-5p
SP9	0,00937725	3	Down	hsa-miR-629-3p / hsa-miR-627-3p / hsa-miR-4685-3p
ABRAXAS2	0,02184939	2	Down	hsa-miR-627-3p / hsa-miR-629-3p
CYYR1	0,02184939	2	Down	hsa-miR-629-3p / hsa-miR-4286
FAM160A1	0,02184939	2	Down	hsa-miR-1538 / hsa-miR-3916
KHSRP	0,02184939	5	Down	hsa-miR-1180-3p / hsa-miR-548e-3p / hsa-miR-3615 / hsa-miR-4286 / hsa-miR-1538
KIF26B	0,02184939	2	Down	hsa-miR-629-3p / hsa-miR-627-3p
TMEM170B	0,02184939	3	Down	hsa-miR-1271-5p / hsa-miR-4685-3p / hsa-miR-3916
TMEM44	0,02184939	3	Down	hsa-miR-629-3p / hsa-miR-627-3p / hsa-miR-4685-3p
TMEM87B	0,02184939	2	Down	hsa-miR-629-3p / hsa-miR-4685-3p
TSKU	0,02184939	4	Down	hsa-miR-4286 / hsa-miR-1271-5p / hsa-miR-4685-3p / hsa-miR-3916

Table S8. Top enriched target genes for specific miRNA deregulation signature in HIVpatients. Here we have included the target gene symbol (Gene_symbol), the adjusted p-value (FDR, corrected p-value for multiple testing performed with the Benjamin–Hochberg procedure), the number of SDE miRNAs that interact with each enriched gene (Num_interactions), the miRNA identifier in miRBase format and its deregulation sense (miRNA_expression).

Gene_symbol	FDR	Num_interactions	miRNAs_expression	ncRNAs
ALG1	0,0263088	3	Up	hsa-miR-4435 / hsa-miR-223-5p / hsa-miR-6852-5p
GLA	0,0263088	2	Up	hsa-miR-769-5p / hsa-miR-4435
HIST1H4C	0,0263088	2	Up	hsa-miR-425-3p / hsa-miR-3150b-3p
NCS1	0,0263088	3	Up	hsa-miR-3150b-3p / hsa-miR-4746-5p / hsa-miR-6852-5p
NELL2	0,0263088	2	Up	hsa-miR-223-5p / hsa-miR-769-5p
XRCC2	0,0263088	3	Up	hsa-miR-769-5p / hsa-miR-4435 / hsa-miR-6852-5p
CKAP2L	0,02911694	2	Up	hsa-miR-769-5p / hsa-miR-3150b-3p
DMXL1	0,02911694	2	Up	hsa-miR-4435 / hsa-miR-223-5p
EDA2R	0,02911694	2	Up	hsa-miR-3150b-3p / hsa-miR-223-5p
GGPS1	0,02911694	2	Up	hsa-miR-769-5p / hsa-miR-6852-5p
CCL16	0,01479768	2	Down	hsa-miR-4516 / hsa-miR-542-3p
PIM1	0,01479768	2	Down	hsa-miR-4516 / hsa-miR-542-3p
SUPT16H	0,01479768	2	Down	hsa-miR-542-3p / hsa-miR-494-3p
TFPI	0,01479768	2	Down	hsa-miR-542-3p / hsa-miR-494-3p
ZADH2	0,01479768	2	Down	hsa-miR-4516 / hsa-miR-542-3p
AKT1	0,01519299	2	Down	hsa-miR-542-3p / hsa-miR-494-3p
BIRC5	0,01612581	2	Down	hsa-miR-542-3p / hsa-miR-494-3p
CDC14B	0,01873976	2	Down	hsa-miR-4516 / hsa-miR-542-3p
EN2	0,01873976	2	Down	hsa-miR-4516 / hsa-miR-494-3p
NF2	0,01873976	2	Down	hsa-miR-4516 / hsa-miR-542-3p
